# Force Distribution Within Spinal Tissues During Posterior to Anterior Spinal Manipulative Therapy: A Secondary Analysis

**DOI:** 10.3389/fnint.2021.809372

**Published:** 2022-02-04

**Authors:** Martha Funabashi, Alexander Cleveland Breen, Diana De Carvalho, Isabelle Pagé, François Nougarou, Martin Descarreaux, Gregory N. Kawchuk

**Affiliations:** ^1^Division of Research and Innovation, Canadian Memorial Chiropractic College, Toronto, ON, Canada; ^2^Chiropractic Department, Université du Québec à Trois-Rivières, Trois-Rivières, QC, Canada; ^3^Faculty of Science and Technoloy, Bournemouth University, Poole, United Kingdom; ^4^Faculty of Medicine, Memorial University of Newfoundland, St. John’s, NL, Canada; ^5^Department of Electrical and Computer Engineering, Université du Québec à Trois-Rivières, Trois-Rivières, QC, Canada; ^6^Human Kinetics Department, Université du Québec à Trois-Rivières, Trois-Rivières, QC, Canada; ^7^Department of Physical Therapy, University of Alberta, Edmonton, AB, Canada

**Keywords:** spinal manipulation, forces, biomechanics, lumbar vertebrae, secondary analysis, porcine

## Abstract

**Background:**

Previous studies observed that the intervertebral disc experiences the greatest forces during spinal manipulative therapy (SMT) and that the distribution of forces among spinal tissues changes as a function of the SMT parameters. However, contextualized SMT forces, relative to the ones applied to and experienced by the whole functional spinal unit, is needed to understand SMT’s underlying mechanisms.

**Aim:**

To describe the percentage force distribution between spinal tissues relative to the applied SMT forces and total force experienced by the functional unit.

**Methods:**

This secondary analysis combined data from 35 fresh porcine cadavers exposed to a simulated 300N SMT to the skin overlying the L3/L4 facet joint via servo-controlled linear motor actuator. Vertebral kinematics were tracked optically using indwelling bone pins. The functional spinal unit was then removed and mounted on a parallel robotic platform equipped with a 6-axis load cell. The kinematics of the spine during SMT were replayed by the robotic platform. By using serial dissection, peak and mean forces induced by the simulated SMT experienced by spinal structures in all three axes of motion were recorded. Forces experienced by spinal structures were analyzed descriptively and the resultant force magnitude was calculated.

**Results:**

During SMT, the functional spinal unit experienced a median peak resultant force of 36.4N (IQR: 14.1N) and a mean resultant force of 25.4N (IQR: 11.9N). Peak resultant force experienced by the spinal segment corresponded to 12.1% of the total applied SMT force (300N). When the resultant force experienced by the functional spinal unit was considered to be 100%, the supra and interspinous ligaments experienced 0.3% of the peak forces and 0.5% of the mean forces. Facet joints and ligamentum flavum experienced 0.7% of the peak forces and 3% of the mean forces. Intervertebral disc and longitudinal ligaments experienced 99% of the peak and 96.5% of the mean forces.

**Conclusion:**

In this animal model, a small percentage of the forces applied during a posterior-to-anterior SMT reached spinal structures in the lumbar spine. Most SMT forces (over 96%) are experienced by the intervertebral disc. This study provides a novel perspective on SMT force distribution within spinal tissues.

## Introduction

Spinal manipulative therapy (SMT) is a conservative intervention commonly used to treat spinal pain and other musculoskeletal conditions (Hurwitz, 2012; Beliveau et al., 2017). It involves the application of a high velocity, low amplitude force to a targeted region of the spine (Triano, 2001; Herzog, 2010). These applied forces mechanically load the spine and surrounding structures, capable of triggering neuromechanical responses which are considered to be related to physiological and clinical effects (Lima et al., 2020; Gevers-Montoro et al., 2021). Forces applied during SMT have been the focus of several studies, primarily to investigate how the input parameters of SMT (e.g., force-time characteristics, application site, etc.) influence the resulting neuromechanical responses (Pasquier et al., 2019; Lima et al., 2020).

The force magnitude of SMT during preload has been reported to influence vertebral displacement, paraspinal muscle activity, and muscle spindle discharge frequency (Nougarou et al., 2014b; Reed et al., 2014a). The total peak force magnitude at the thrust phase has also been observed to influence vertebral displacement and acceleration, paraspinal muscle activity, and mechanical thresholds of lateral thalamic nociceptive specific neurons (Keller et al., 2003, 2006; Colloca et al., 2004, 2006; Nougarou et al., 2014a; Reed et al., 2014b). Similarly, the SMT thrust amplitude (displacement) has been reported to influence muscle spindle discharge frequency (Pickar et al., 2007). The interaction between SMT force amplitude and duration significantly influenced spinal stiffness and muscle spindle discharge frequency (Pickar et al., 2007; Vaillant et al., 2012; Cao et al., 2013). SMT thrust duration was shown to influence vertebral displacement and acceleration, paraspinal muscle activation and muscle spindle discharge frequency (Colloca et al., 2006; Pickar and Kang, 2006; Pagé et al., 2014). SMT thrust loading rate has also been observed to influence vertebral displacements and muscle spindle response (Reed et al., 2013; Nougarou et al., 2016). In addition to these force-time characteristics, other input parameters, such as location of applied force has also been investigated and reported to influence vertebral displacements, spinal stiffness, paraspinal muscle activity and muscle spindle discharge frequency (Colloca and Keller, 2001; Keller et al., 2003; Colloca et al., 2004; Edgecombe et al., 2013; Reed et al., 2015; Reed and Pickar, 2015).

Previous biomechanical studies have investigated the loading of spinal structures during SMT application and how forces are distributed among them. Specifically, the intervertebral disc has been shown to experience the greatest forces during SMT (Kawchuk et al., 2010; Funabashi et al., 2016). Additionally, the distribution of SMT forces among spinal tissues has been shown to change as a function of the applied SMT parameters, such as location at which SMT is applied and method of SMT application (Funabashi et al., 2017a,b, 2018).

Although the absolute force magnitudes experienced by spinal structures during SMT have been reported (Kawchuk et al., 2010; Funabashi et al., 2016, 2017a,b,^2018^), the detailed description of the SMT force distribution within spinal structures, presented as percentages of the total applied force, has not been published. Such description would put the magnitude of forces experienced by the spinal segment and each spinal structure into context relative to applied SMT and the whole functional spinal unit, respectively. This, in turn, can have significant impact on advancing our knowledge regarding SMT’s underlying mechanisms and may help inform future clinical investigations. Specifically, preferential loading of certain spinal structures as a function of the SMT input parameters may have an influence on SMT’s neuromechanical effects. Consequently, clarifying the proportion of applied SMT forces that reach spinal tissues, and their respective distribution, will advance our understanding of the underlying mechanisms of SMT and potentially improve the effectiveness and safety of SMT.

Therefore, the aim of this study was to describe the SMT force distribution between spinal tissues in terms of percentage of the total applied SMT force, and the total force experienced by the spinal segment.

## Materials and Methods

This was a secondary analysis of the forces experienced by spinal structures during a standardized posterior-to-anterior SMT application. A subset of data from three previous studies with similar experimental procedures were combined (Funabashi et al., 2017a,b, 2018). The first study (*n* = 10) investigated the potential interaction between SMT force magnitude and location of application, applying 100N, 300N, and 500N SMTs at the skin overlying the left L3/L4 facet joint and L4 transverse process (Funabashi et al., 2017a). The second study (*n* = 13) investigated the change in SMT force distribution among spinal structures when a 300N SMT was applied at different locations: the skin overlying L2/L3 and L3/L4 facet joints, L3 and L4 transverse processes and the interspace between those facet joints and transverse processes (Funabashi et al., 2018). The third study (*n* = 12) compared the forces experienced by spinal tissues when different methods were used to apply an SMT to the skin overlying the left L3/L4 facet joint (Funabashi et al., 2017b).

**FIGURE 1:**
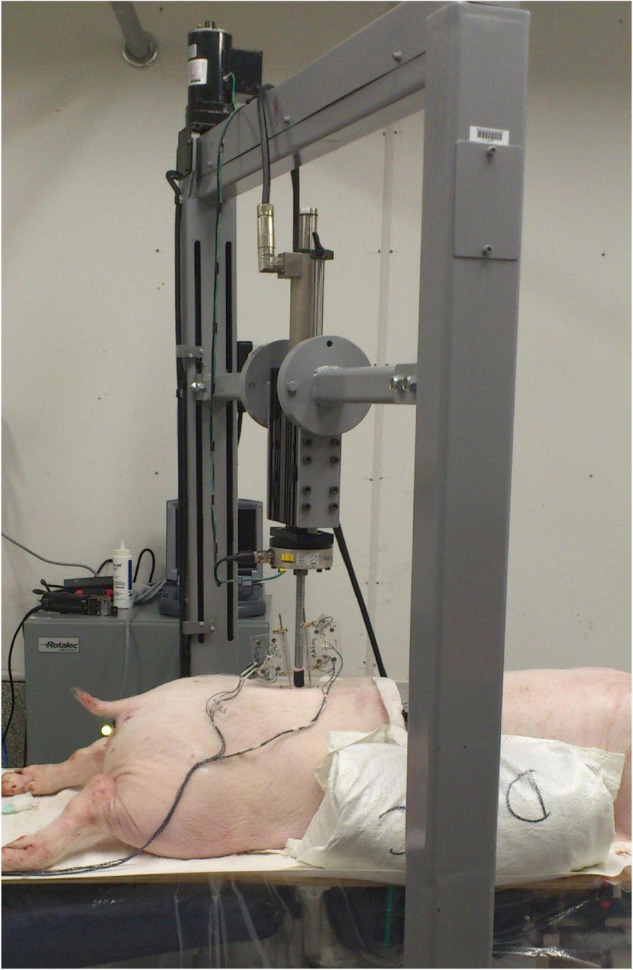
Experimental set up: porcine cadaveric model with rectangular flags with four infrared light-emitting diode markers attached to bone pins drilled into L3 and L4 vertebrae and the mechanical device with a servo-controlled linear motor actuator to apply the standardized spinal manipulative therapy

**FIGURE 2:**
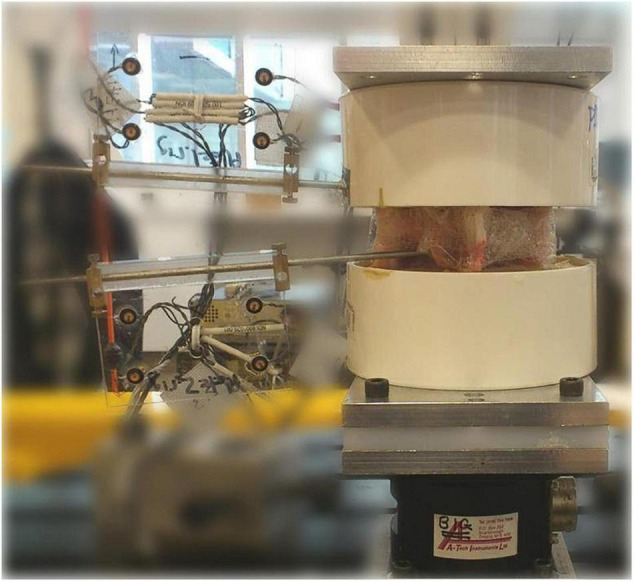
Potted specimen with L4 (bottom pot) fixed to the 6-axis load cell and L3 (upper pot) fixed to a stationary cross beam.

Thus, this secondary analysis includes data from 35 fresh porcine cadaveric models [Duroc × (Large White × Landrace breeds)] of approximately 60–65 kg who received a standardized SMT simulation (30 N preload, 300 N peak force, time to peak of 112.5 ms and consequent 2.6 N/ms loading rate) to the skin overlying the left L3/L4 facet joint applied by a mechanical device using a servo-controlled linear motor actuator (Descarreaux et al., 2013; Figure 1). The detailed methods from each study have been previously reported, but in brief, the three-dimensional SMT vertebral kinematics for each specimen were tracked optically using bone pins drilled into L3 and L4 vertebral bodies with attached infrared light-emitting diode markers (Optotrack Certus, NDI, Waterloo, Canada) at a rate of 400 Hz with a 0.01 mm system resolution and a 0.15 mm rigid body resolution. After SMT application, the L3/L4 functional spinal unit was removed *en bloc*, cleaned of non-ligamentous tissues, and potted in a vertical orientation using dental stone (Modern Materials, South Bend, IN, United States). The caudal end of the specimen was then fixed to a 6-axis load cell (AMTI MC3A-1000, Advanced Mechanical Technology, Inc., Watertown, MA, United States), which was mounted rigidly to a parallel robot platform (Parallel Robotics Systems Corp., Hampton, NH, United States) (Figure 2) with the anatomical axes of the specimen aligned with both the load cell and the robot axes: x = mediolateral (positive in the left direction), y = anterioposterior (positive in the posterior direction), and z = superioinferior (positive in the superior direction). The cranial end of the specimen was fixed to a stationary cross beam and, following the procedures described by Goldsmith et al. (2015), the marker movements caused by SMT were transformed into robot trajectories that replicated the relative motions between L3 and L4 vertebrae recorded by the optical tracking system (Goldsmith et al., 2015). The SMT trajectories were then applied by the robot and forces experienced by the spinal segment were recorded by the load cell. For all specimens, 3 pre-conditioning trials were completed prior to testing and data collection.

Following application of SMT robotic trajectories in the intact specimen, spinal structures were then removed and/or transected and the same robotic trajectories repeated. In this way, the loading distribution within specific spinal tissues was quantified. In all studies, the following spinal structures were removed/transected (via scalpel unless otherwise noted) in the same order for all specimens: (1) supraspinous and interspinous ligaments, (2) bilateral facet capsules, posterior facet joints (via rongeur) and ligamentum flavum, (3) intervertebral disc and anterior and posterior longitudinal ligaments. By using serial dissection, peak (maximum force during thrust) and mean (average force during preload and thrust) forces induced by the simulated SMT experienced by spinal structures in all three axes of motion were recorded and analyzed descriptively. As the objective of this study was to describe the SMT force distribution between spinal tissues in terms of percentage relative to the applied SMT force and the total forces experienced by the spine segment, the resultant force magnitude (F_res_) was calculated using Equation 1, where Fx corresponds to the force in the mediolateral direction, Fy is the force in the anterioposterior direction and Fz is the force in the superioinferior direction. Percentage of peak force experienced by the intact specimen was calculated in reference to the applied SMT force (i.e., the applied SMT peak force [300N] was considered 100%), and percentages of peak and mean forces experienced by each spinal tissue was calculated in reference to the those experienced by the intact specimen (i.e., the peak and mean forces experienced by the intact specimen were considered 100%).


(1) $$F_{\text{res}}=\sqrt{(F_{x}^{2}+F_{y}^{2}+F_{z}^{2}})$$

## Results

### Vertebral Rotations

Mean L4 vertebral rotations relative to L3 created in the intact specimens at peak loads during the application of a posterior-to-anterior SMT were 1.33° (± 1.31) in the x-axis (flexion-extension), −0.57° (± 1.02) in the y-axis (lateral bending), and −0.94° (± 0.96) in the z-axis (axial rotation).

### Forces

Force data presented a non-parametric distribution, therefore, descriptive statistics are presented as median and interquartile range (IQR). Table 1 presents the median and IQR peak forces in all three axes of motion experienced by the intact specimens and normalized relative peak forces experienced by spinal structures during a posterior-to-anterior SMT. Similarly, median mean forces in all three axes of motion experienced by the intact specimens and normalized relative mean forces experienced by spinal structures are presented in Table 2. Resultant force magnitude of intact specimen and spinal tissues as well as percentage forces of spinal structures relative to the intact specimen condition are also presented in Tables 1, 2.

**TABLE 1:** Peak forces [median (IQR)] experienced by the intact specimens and normalized relative peak forces experienced by spinal structures.

Spinal structure	Fx [N]	Fy [N]	Fz [N]	Resultant force vector magnitude [N]	Relative to intact specimen
Intact specimen	−17.97 [13.12]	−24.18 [16.30]	23.29 [25.77]	36.4 [14.1]	100%
Supra- and interspinous ligaments	0.03 [0.04]	0.02 [0.05]	0.00 [0.08]	0.1 [0.1]	0.3%
Facet joints, capsules and ligamentum flavum	−0.05 [0.37]	−0.17 [0.31]	0.00 [0.20]	0.3 [0.2]	0.7%
Intervertebral disc, anterior and posterior longitudinal ligaments	−17.86 [13.10]	−24.15 [16.65]	23.08 [27.37]	36.0 [14.2]	99.0%

*IQR, interquartile range; F, force; x, mediolateral direction (positive in the left direction); y, anterioposterior direction (positive in the posterior direction); z, superioinferior direction (positive in the superior direction); N, Newtons.*

**TABLE 2:** Mean forces [median (IQR)] experienced by the intact specimens and normalized relative mean forces experienced by spinal structures.

Spinal structure	Fx [N]	Fy [N]	Fz [N]	Resultant force vector magnitude [N]	Relative to intact specimen
Intact specimen	0.22 [14.48]	−5.23 [6.95]	23.54 [9.97]	26.6 [11.9]	100%
Supra- and interspinous ligaments	0.06 [0.08]	0.08 [0.10]	0.02 [0.18]	0.2 [0.1]	0.5%
Facet joints, capsules and ligamentum flavum	0.24 [0.55]	−0.24 [0.41]	0.02 [0.26]	0.8 [0.3]	3.0%
Intervertebral disc, anterior and posterior longitudinal ligaments	1.03 [14.76]	−5.18 [7.65]	23.53 [10.37]	25.6 [14.2]	96.5%

*IQR, interquartile range; F, force; x, mediolateral direction (positive in the left direction); y, anterioposterior direction (positive in the posterior direction); z, superioinferior direction (positive in the superior direction); N, Newtons.*

As shown in Table 1, the intact functional spinal unit experienced a median peak resultant force magnitude of 36.4N (IQR: 14.1N), which corresponds to 12.1% of the total peak force that was applied during a posterior-to-anterior SMT (300N).

## Discussion

This is the first study to describe the SMT force distribution within spinal structures in terms of percentage relative to the total applied SMT force and the total force experienced by the functional spinal unit. This comprehensive description of SMT force distribution may not only contribute to investigations of the mechanisms underlying this conservative intervention, but also guide training and education of SMT as well as future potential development of techniques to preferentially load specific spinal structures.

The vertebral motion of the L3/L4 segment indicates that at maximum load, the L4 vertebral body was in an average of 1.33° extension, 0.57° right lateral bend and 0.94° right axial rotation in relation to L3. Given the posterior-to-anterior SMT application to the left side of the spine, the observed vertebral motion was expected and in agreement with previous studies that have described vertebral movement during SMT (Gal et al., 1997; Kawchuk et al., 2010). Specifically, Kawchuk et al. (2010) reported similar vertebral motions to the current study during a manual SMT application in a similar model with average extension rotations of 1.96°, lateral bending of 0.61° and axial rotation of 0.45°. Additionally, Gal et al. (1997) observed extension rotations of 0.2°–1.8° and axial rotations of 0.4°–1.2° during manual SMT application at the low thoracic region of human cadavers. Although the vertebral motion magnitude can vary based on the force-time characteristics of the applied SMTs (Herzog et al., 1993; Forand et al., 2004), this provides additional evidence that a complex three-dimensional vertebral movement occurs in spinal segments during SMT application. Importantly, these findings align with previous studies and provides additional evidence that vertebral motion during a posterior-to-anterior SMT remains within normal ranges of motion reported in the literature (Kozanek et al., 2009; Li et al., 2009; Passias et al., 2011; Widmer et al., 2019).

Our results show that it is only a small percentage of the applied SMT forces that reaches spinal structures [median peak force 36.4N (14.1 IQR) of applied 300N] and that, among these, the majority of force is experienced by the intervertebral disc, which is in accordance with previous studies (Kawchuk et al., 2010; Funabashi et al., 2016, 2017a,b,^2018^). This may be related to the intervertebral disc’s contribution to resisting anteroposterior and lateral vertebral motions (Lu et al., 2005; Okushima et al., 2006). Additionally, Schmidt et al. (2007) observed that the combination of lateral bending with extension increased the intervertebral disc’s maximum shear strains, and that the combination of lateral bending with axial rotation, and axial rotation with extension increased the intervertebral disc’s posterolateral fiber strains (Schmidt et al., 2007). Given that a combination of all these motions occur during SMT, it is possible that the increased shear and fiber strains might be potentially related to the loading experienced by the intervertebral disc during SMT. Future biomechanical studies should investigate the specific intervertebral disc’s shear and strains during SMT to further elucidate the SMT effects on this structure.

Conversely, this secondary analysis showed that supraspinous and interspinous ligaments experienced the least forces during posterior-to-anterior SMT. This was not surprising as posterior-to-anterior SMT was observed to move the spinal unit into extension and previous studies have described the supraspinous and interspinous ligaments’ mechanical function to either restrain flexion (especially toward the end of flexion range on motion) (Hindle et al., 1990; Gillespie and Dickey, 2004), or anchor tendons and fascia (Aspden et al., 1987; Hukins et al., 1990). Of note, caudal forces experienced by these ligaments have been observed during SMT (Funabashi et al., 2018), which may be potentially associated with resisting compression during the extension SMT movement (Heuer et al., 2007a,b). Therefore, future studies should investigate the loading of spinal structures during other SMT techniques, such as side-posture SMT, to further elucidate the forces withstood by spinal ligaments.

Based on this study’s results, the greatest SMT loads are experienced by the intervertebral disc, however, these loads are still lower than the ones the disc experiences during passive axial rotation (Funabashi et al., 2016). In the literature, intradiscal pressure has been previously investigated during SMT (Lisi et al., 2006) and during normal activities of daily living, such as sitting and walking (Wilke et al., 1999). Despite the limitations of being two different studies with distinct methodologies, the reported intradiscal pressure during SMT was comparable to the intradiscal pressure reported during activities of walking and sitting combined with flexion, and lower than the pressures reported during sit to stand and lifting 20 kg (Wilke et al., 1999; Lisi et al., 2006). This indicates that although the intervertebral disc experiences the greatest loads during SMT, these are similar to the loads and pressure experienced by the lumbar spine during daily functional activities. However, given the viscoelastic property of biological tissues and the high-velocity dynamic nature of SMT thrust, it is possible that other SMT characteristics (such as loading rate and thrust speed) could elicit different intradiscal pressure and loading of the intervertebral disc. Importantly, patients with lumbar disc herniation have been observed to significantly improve their pain level and disability following SMT (Leemann et al., 2014; Shokri et al., 2018). This suggests that, in addition to changes in intradiscal pressure, SMT may elicit other intervertebral disc responses, which may contribute to SMT’s clinical effects. Indeed, previous studies have reported changes in water disc diffusion following SMT (Beattie et al., 2014; Wong et al., 2019), which has been described to influence the mechanical behavior of the intervertebral disc (Bhattacharya and Dubey, 2020) and could potentially contribute to SMT’s clinical outcomes. Nevertheless, further studies are needed to fully elucidate the underlying mechanisms of SMT and its effects on the intervertebral disc.

### Clinical Implications

Although it has been described that SMT application characteristics significantly change the magnitude of loads experienced by each spinal structure (Kawchuk et al., 2010; Funabashi et al., 2016, 2017a,b,^2018^), results from this secondary analysis show that the intervertebral disc experiences over 96% of the forces that reach the spinal segment during SMT. Future research is needed to fully understand the clinical effects of SMT loading on the intervertebral disc. Additionally, given that a small percentage of the forces applied during a posterior-to-anterior SMT reaches spinal structures, future investigations are needed to quantify the loading of structures adjacent to the spine during this intervention.

### Strengths and Limitations

This study combined several studies of similar standardized methodology to generate a more conclusive dataset regarding the distribution of SMT forces amongst spinal tissues, allowing results to be more robust. While anatomical differences between porcine models and humans are limitations, animal models play an important role in advancing our knowledge and understanding of SMT force distribution and its underlying effects for clinical application (Daly et al., 2016). To quantify loads experienced by discrete spinal tissues, all studies included in this secondary analysis underwent serial dissection removing spinal tissues in the same order in all specimens and, therefore, results are specific to this specific sequence of tissue removal (Funabashi et al., 2015). A different order of tissue removal may affect the loads within each spinal tissue, but not the loads observed for the intact specimen. Results from this analysis are specific to the mechanical posterior-to-anterior 300N SMT and other SMT techniques and force characteristics may present unique loading characteristics of spinal tissues. Additionally, studies included in this secondary analysis were conducted with healthy spine models, limiting the application of our results to pathological conditions. Differences between *in vivo* and *in vitro* conditions such as physiological and muscular effects limit the application of these results to living conditions. Finally, repeated robotic replication of SMT kinematics may potentially have affected the mechanical behavior of cadaveric tissues, so caution should be taken when interpreting our results.

## Conclusion

In this animal model, 12.1% of the total peak forces applied during a mechanical, unilateral, posterior-to-anterior SMT reached spinal structures in the lumbar spine. Most SMT forces reaching spinal structures (over 96%) are experienced by the intervertebral disc. This study provides a novel perspective on SMT force distribution within spinal tissues, putting the magnitude of forces experienced by the spinal segment and each spinal structure into context relative to applied SMT and the whole functional spinal unit, respectively. These findings can be used to inform SMT training when using force-sensing technologies and guide future mechanistic investigations.

## Data Availability Statement

The raw data supporting the conclusions of this article will be made available by the authors upon request, without undue reservation.

## Ethics Statement

Ethical review and approval was not required for the animal study because this was a secondary analysis of data. All primary studies reporting original data state that experimental protocols were approved by the Animal Care and Use Committee of the University of Alberta.

## Author Contributions

MF, AB, DD, and IP contributed to the study conceptualization. MF, AB, DD, IP, FN, MD, and GK contributed substantially to various aspects of the acquisition of original data, data analysis, and interpretation. MF drafted the manuscript. AB, DD, IP, FN, MD, and GK substantially revised it. All authors significantly contributed to the manuscript and approved the submitted version.

## Conflict of Interest

The authors declare that the research was conducted in the absence of any commercial or financial relationships that could be construed as a potential conflict of interest.

## Publisher’s Note

All claims expressed in this article are solely those of the authors and do not necessarily represent those of their affiliated organizations, or those of the publisher, the editors and the reviewers. Any product that may be evaluated in this article, or claim that may be made by its manufacturer, is not guaranteed or endorsed by the publisher.
